# Positive inotropic effects of glucose-dependent insulinotropic polypeptide in the human atrium and the mouse atrium

**DOI:** 10.1007/s00210-025-04485-1

**Published:** 2025-07-26

**Authors:** J. Neumann, B. Hofmann, U. Gergs

**Affiliations:** 1 Institute for Pharmacology and Toxicology, Medical Faculty, Martin Luther University Halle-Wittenberg, Magdeburger Straße 4, 06097 Halle (Saale), Germany; 2 Department of Cardiac Surgery, Mid-German Heart Centre, University Hospital Halle, Ernst-Grube-Straße 40, 06097 Halle (Saale), Germany

**Keywords:** GIP, GIP receptor, Human atrium, Mouse atrium

## Abstract

Glucose-dependent insulinotropic polypeptide formerly called gastrin inhibitory peptide (GIP), a peptide composed of 42 amino acids, is formed in duodenal and jejunal cells. GIP acts via GIP receptors (GIPR). GIPR can stimulate adenylyl cyclases (AC) and increase intracellular cyclic adenosine-3´,5´-monophosphate (cAMP) levels. The physiological role of GIPR in the human heart is not fully understood. Thence, force of contraction (FOC) was studied in isolated electrically driven (1 Hz) human right atrial preparations from patients undergoing bypass surgery due to severe coronary heart disease. We noted that in paced human atrium, GIP increased FOC. This effect was reduced by a GIPR-antagonist (ProGIP). In the presence of 0.1 µM cilostamide, a phosphodiesterase (PDE) 3 inhibitor, the positive inotropic effects (PIE) of GIP were more potent and efficient to raise FOC. Up to 100 nM GIP failed to heighten the spontaneous beating rate in mouse right atrial preparations, but increased FOC in electrically driven left atrial mouse preparations but only in the presence of a PDE 4 inhibitor (100 nM rolipram). We conclude that the human atrium and the mouse atrium contain functional GIPR with respect to FOC.

## Introduction

Human gastrin inhibitory peptide (GIP) is a peptide composed of 42 amino acids (Alaña et al. 2007). GIP is generated mainly in the so-called enteroendocrine “K-cells” of the duodenum and jejunum (Heimbürger et al. 2020). GIP is liberated from these intestinal stores when the K-cells of the intestine come into contact with carbohydrates, proteins or lipids when we eat (Jorsal et al. 2018). Released intestinal GIP enters the plasma of humans and thereby reaches the pancreas, the brain but also the heart (Jorsal et al. 2018). GIP potentiates the release of insulin from β-cells in the islets of Langerhans via GIPR in the pancreas (Mroz et al. 2019, Hammoud and Drucker 2023).

GIPR, at least as mRNA, were found in mouse whole hearts, more specifically also in mouse ventricular sections, in mouse atrial sections, in neonatal mouse cardiomyocytes, in adult mouse cardiomyocytes and in surgical samples from human left atria, human right atria, human left ventricles and human right ventricles (Baggio et al. 2018, Ussher et al. 2018, Hiromura et al. 2016).

In HL-1 atrial cardiomyocytes (a rat tumor line) and in isolated neonatal mouse cardiomyocytes 10 nM GIP elevated intracellular cAMP levels (Hiromura et al. 2016, Ussher et al. 2018). In addition, in cardiomyocytes GIPR has stimulated cGMP formation and activated ERK and MEK (Ussher et al. 2018). Hence, like isoprenaline, a positive inotropic drug, which elevated cardiac cAMP via β-adrenoceptors, GIP might increase cardiac FOC.

To the best of our knowledge, a direct inotropic effect of GIP in the human atrium has not yet been described. For comparison, we also studied cardiac effects of GIP in mouse atrial preparations. The mouse atrium was studied because mice are at present widely used in gene inactivation studies: e.g. GIPR has been deleted in mice and the cardiac functions in these mice were investigated (Ussher et al. 2018).

Therefore, we tested mainly the following hypotheses:

1. Does GIP act via GIPR in human atrium?
2. Does GIP increase FOC in mouse left atrium or beating rate in mouse right atrium?
3. Does GIP augment FOC in human atrium?

Parts of this study have been published in an abstract form (Neumann et al. 2024).

## Materials and methods

### Contractile studies in mice

Housing, handling, raising and sacrifice of mice complied with local regulations. Mice were subjected to cervical dislocation before the thorax was opened. The heart as a whole was prepared and moved to a glass Petri dish filled with a modified Tyrode’s solution, kept at room temperature. In this buffer, the right or left atrial preparations from the mice (wild type: CD1, random sex, about 120 days of age) were isolated. Then mouse left atria and mouse right atria were mounted in organ baths (containing 10 ml buffer volume) as previously described (e.g. Gergs et al. 2017). The modified Tyrode’s solution contained in millimolar concentrations (mM): 119.8 NaCI, 5.4 KCI, 1.8 CaCl_2_, 1.05 MgCl_2_, 0.42 NaH_2_PO_4_, 22.6 NaHCO_3_, 0.05 Na_2_EDTA, 0.28 ascorbic acid and 5.05 glucose. Ascorbic acid is used here as an antioxidant to maintain the activity of, for instance, isoprenaline. The solution was continuously gassed with 95% O_2_ and 5% CO_2_ and maintained at 37 °C and pH 7.4 in the organ baths. Spontaneously beating mouse right atria were used to study any chronotropic effects. Mouse left atria were used to study force under isometric conditions. Mouse left atria were stretched to optimal length that allowed maximal generation of FOC (3–4 mN). Mouse left atria were stimulated (60 beats per minute, bpm) electrically with platinum electrodes with rectangular impulses of direct currents from a Grass stimulator SD 9 (Ohio, USA). Voltage ranged between 5 and 10 V, just sufficient to initiate contractions. Electrical impulses had a length of 5 ms. The signals from the force transducer were fed into a bridge amplifier, digitized and stored on a commercial personal computer. The signals were quantified using a commercial software (LabChart 8® from ADInstruments bought through their distributor in Oxford, England). Using LabChart® we calculated beating rate, force of contraction in mN, time to peak tension, time of relaxation, rate of tension development and rate of tension relaxation.

### Contractile studies on human preparations

The contractile studies on human atrium were performed using the same setup and modified Tyrode’s solution as used for mouse preparations described in the preceding paragraph. Our methods used for atrial contraction studies in human samples have been previously published and were not altered in this study (e.g. Gergs et al. 2009, 2017). In brief, human right atrial preparations obtained during the cardiac surgery at the sites where extracorporeal circulation needles were inserted were rapidly transferred into the laboratory in modified Tyrode’s solution. Samples were cut into small trabecular muscle pieces. These muscle strips were then mounted under isometric conditions with metal hooks at each end of the muscle in a glass organ bath. Like mouse left atrium (vide supra) also human muscle strips were electrically stimulated at 1 Hz with rectangular impulses of 5 ms duration and 10% above the voltage required for initiation of the beating (around 10 V). Human muscle strips were stretched to the maximum of the force-contraction relationship. Signals were amplified and quantified as described above for mouse left atrium. The human atria were obtained from male patients of 58–80 years of age. The patients suffered from severe coronary diseases (two and three vessel diseases). The cardiac drug therapy included acetylsalicylic acid, apixaban, furosemide and metoprolol. Cardiac comorbidities included in addition to angina pectoris also hypertension and atrial fibrillation. In some experiments, we finally applied receptor antagonists to the organ baths and then the β-adrenoceptor agonist isoprenaline as a positive control, as delineated in the figure legends.

As described in the appropriate legends, in mouse atria and human atrium, GIP was applied at one concentration or cumulatively. This occurred without or after pre-incubation with 100 nM rolipram (mouse atria) or 1 µM cilostamide (human atria). Finally, where indicated the GIPR antagonist ProGIP (Gault et al. 2003) or isoprenaline were added.

Similarly as in mouse atrial preparations, we calculated with LabChart®, force of contraction in mN, time to peak tension, time of relaxation, rate of tension development and rate of tension relaxation in HAP.

### Data analysis

Data shown are mean ± standard error of the mean. Statistical significance was estimated using Student’s *t* test or the analysis of variance followed by Bonferroni correction for multiple comparisons as described in the legends. A *p* value < 0.05 was considered to be significant.

### Drugs and materials

**Fig. 1:**
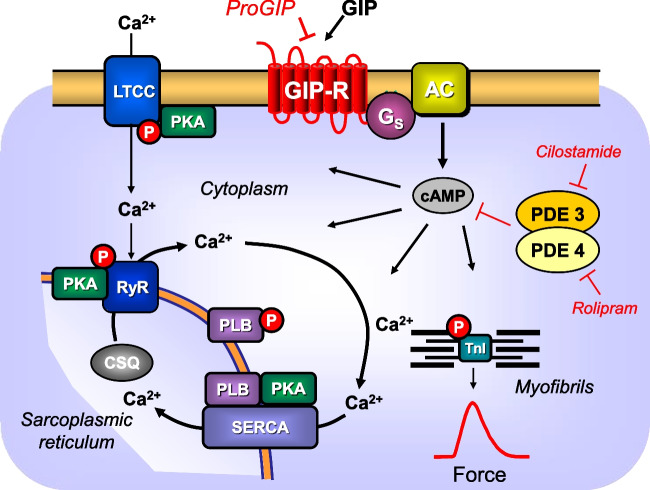
Schema of GIP action in the mammalian heart. Putative mechanism(s) of action of GIP in cardiomyocytes. GIP stimulates GIP receptors (GIPR) and ProGIP is a GIPR antagonist. Then via stimulatory GTP-binding proteins (Gs), adenylyl cyclases (AC) catalyzed the formation of cAMP. This cAMP activates a cAMP-dependent protein kinase (PKA). PKA is compartmentalized. This PKA activates by phosphorylation (P) cardiac regulatory proteins. The cAMP is degraded by phosphodiesterases (PDE 3 in human hearts and PDE 4 in mouse hearts) that can be inhibited by cilostamide or rolipram, respectively. PKA can phosphorylate the inhibitory subunit of troponin (TnI). SERCA pumps Ca^2+^ into the sarcoplasmic reticulum. Ca^2+^ binds to calsequestrin. RyR indicates the ryanodine receptor. LTCC means the L-type Ca^2+^ channel. Myofibrils are responsible for the generation of force which is symbolized here by a single muscle contraction tracing over time. Red circles symbolize phosphorylation of target proteins

(-)Isoprenaline tartrate was from Sigma-Aldrich (Taufkirchen, Germany). Cilostamide and Pro3GIP (ProGIP, article number 5838/1) were from Tocris (Wiesbaden, Germany). Human GIP (article number 4030658) came from Bachem (Bubendorf, Switzerland). All other chemicals were of the highest purity grade commercially available. Deionized water was used throughout the experiments to prepare a modified Tyrode’s solution. Stock solutions were prepared fresh daily (Fig. 1).

## Results

**Fig. 2:**
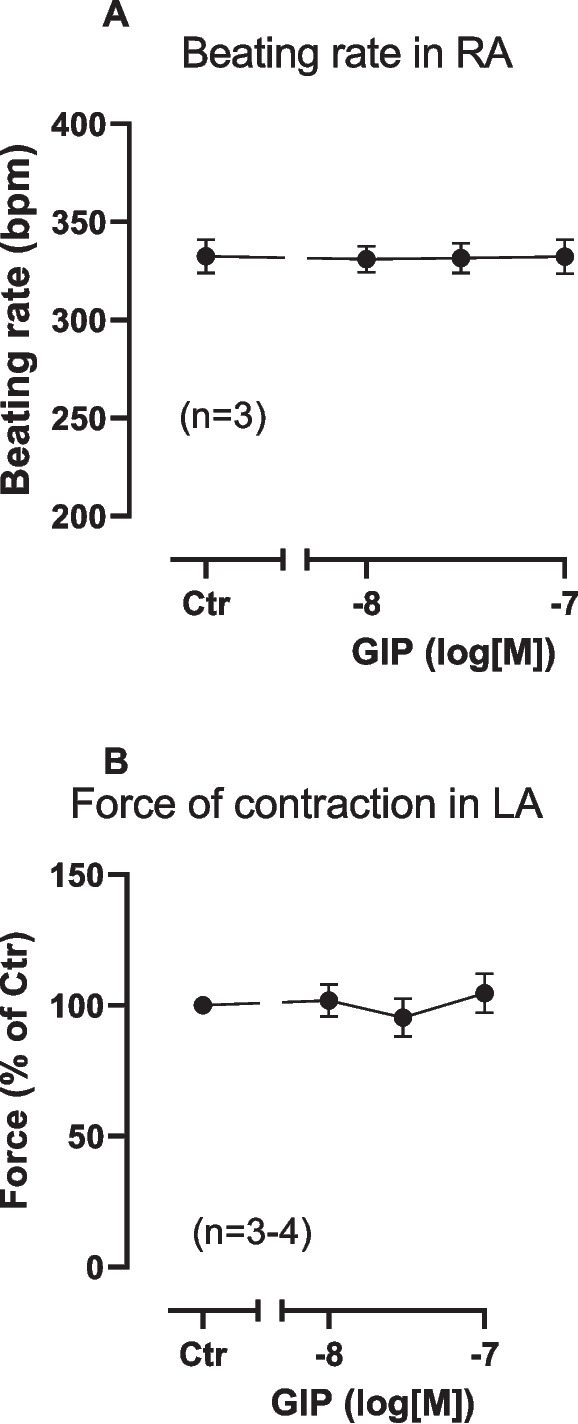
GIP alone fails to increase FOC or beating rate in mouse left atrium. Concentration-dependent effect of GIP alone on beating rate in mouse right atrium (**A**) and on FOC in mouse left atrium (**B**). Ordinate in **A** indicates beating rate in beats per minute (bpm). Ordinate in **B** indicates force in percentage of pre-drug value (Ctr before GIP). Numbers in brackets give numbers of experiments. * indicate *p* < 0.05 vs. Ctr

**Fig. 3:**
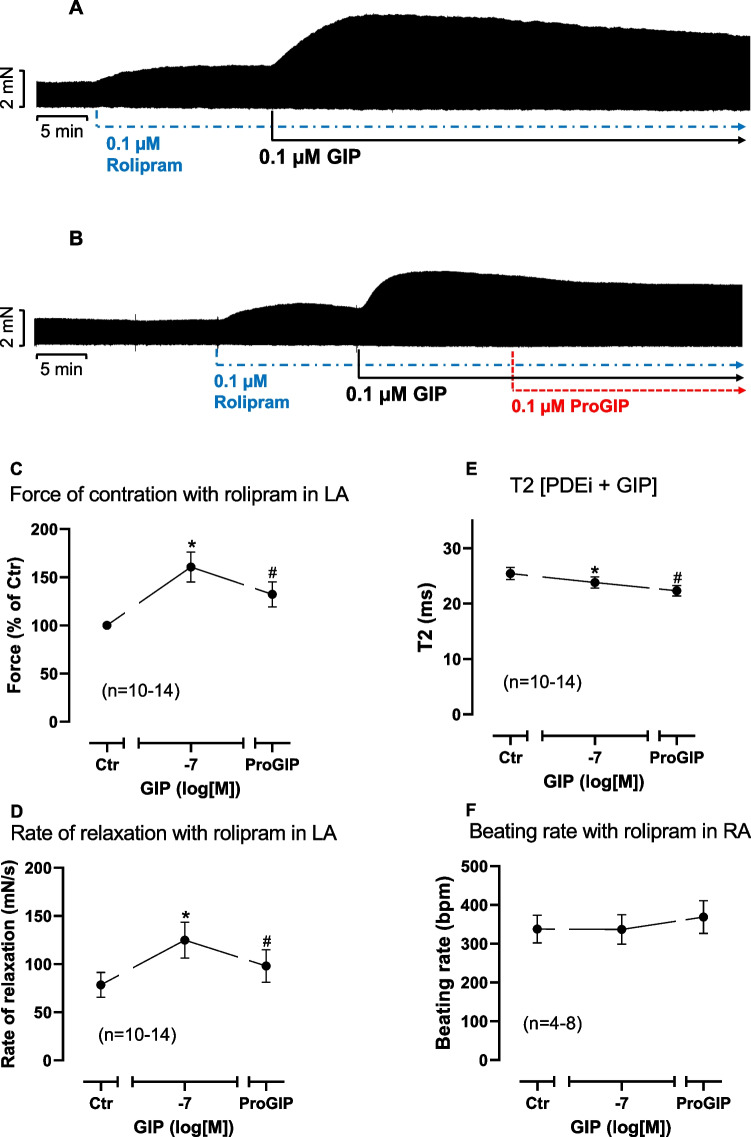
GIPR antagonist decreases GIP stimulated FOC in the presence of rolipram in mouse left atrium. Original recordings of two different experiments (**A** and **B**): first rolipram thereafter GIP was applied. Then the GIPR antagonist ProGIP was added. In both muscle strips, rolipram alone increased FOC and then added GIP increased FOC further. Additionally applied ProGIP reduced FOC partially in a time-dependent manner. **C**: Summarized data on FOC when first rolipram (Ctr) and thereafter GIP and then ProGIP were applied in percentage of the value immediately before GIP addition. **D**: Summarized data on rate of tension relaxation when first rolipram and thereafter GIP and then ProGIP were applied in milli Newton per second (mN/s). **E**: Summarized data on time of relaxation when first rolipram and thereafter GIP and then ProGIP were applied in milliseconds (ms). **F**: Summarized data on beating rate in mouse right atrial preparations when first rolipram (Ctr) and thereafter GIP and then ProGIP were applied in beats per minute (bpm). Horizontal bars indicate time in minutes (min). Vertical arrows indicate the time of drug addition to the organ bath. Abscissae indicate values after rolipram (Ctr) or in the presence of 100 nM GIP alone or 100 nM GIP and 100 nM ProGIP. Numbers in brackets give numbers of experiments. * indicate *p* < 0.05 vs. Ctr or GIP as appropriate

In mouse right atrium up to 100 nM GIP alone failed to increase the beating rate (Fig. 2A). Likewise in mouse left atrium, GIP alone failed to heighten FOC (Fig. 2B). However, in the presence of 100 nM rolipram (a PDE 4 inhibitor), GIP exerted positive inotropic effects (PIE). We gave rolipram to increase force of contraction by inhibition of phosphodiesterase (PDE) 4. We used 100 nM rolipram, a concentration that slightly increased FOC based on our previous studies (e.g. Neumann et al. 2021). This is depicted in an original recording (Fig. 3A). These PIE were attenuated by the GIP antagonist ProGIP. An original tracing for these findings is demonstrated in Fig. 3B. Several such experiments are summarized in Fig. 3C in milli Newton (mN). This effect was antagonized by 100 nM ProGIP, a mutated GIP derivative that acts as a GIPR antagonist in this concentration (Gault et al. 2003). GIP augmented FOC as can be seen in Fig. 3C. Likewise, in the presence of rolipram, GIP increased the rate of muscle tension relaxation. This can be seen in absolute values in Fig. 3D. At the same time GIP in the presence of rolipram shortened time of relaxation (Fig. 3E).

However, in mouse right atrium up to 100 nM GIP even in the presence of 100 nM rolipram failed to induce a positive chronotropic effect as depicted in Fig. 3F in absolute values.

**Fig. 4:**
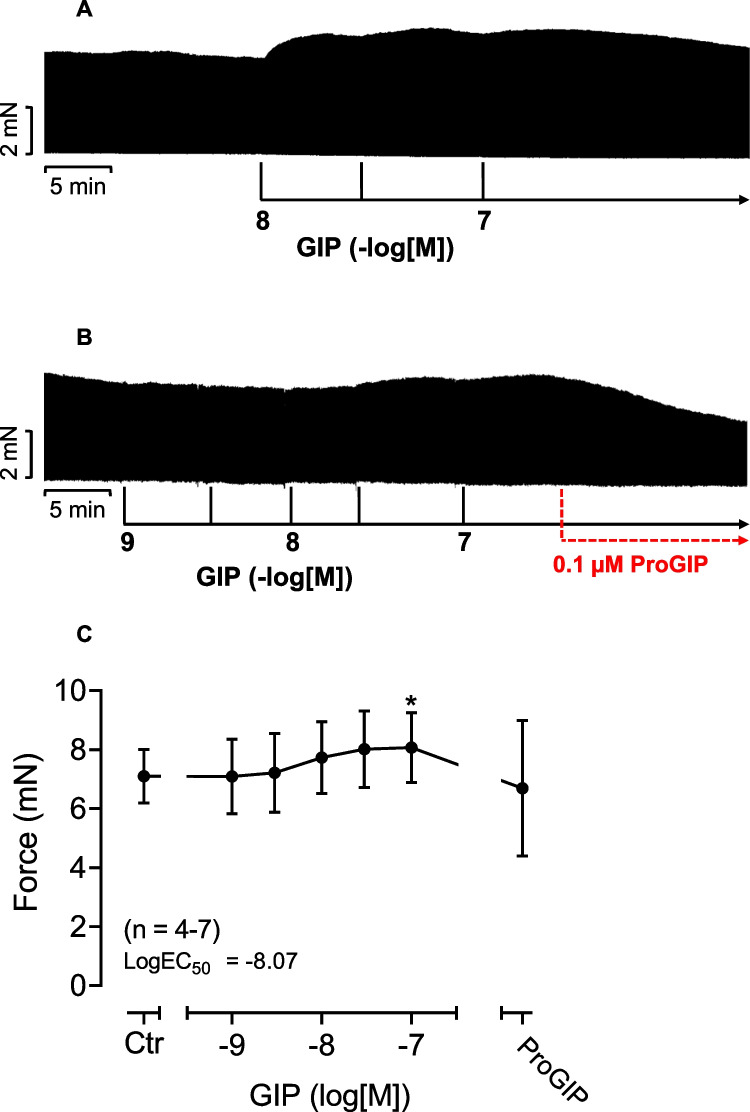
GIP alone increases FOC in human atrium and is antagonized by [Pro3]-GIP. A: Original recordings of two different experiments (**A** and **B**): GIP was applied (in both **A** and **B**) to human atrium. Then the GIPR antagonist ProGIP was added in **B**. In both muscle strips, GIP increased FOC in a concentration and time-dependent manner. Additionally applied 0.1 µM ProGIP reduced FOC in a time-dependent manner (**B**). **C**: concentration-dependent effects of GIP alone or after subsequently applied ProGIP on force of contraction in mN. Ordinates indicate force of contraction in human atrium in milli Newton (mN, **A**, **B** and **C**). Horizontal bars indicate time in minutes (min). Vertical arrows indicate the time of drug addition to the organ bath. Abscissae indicate pre-drug values (Ctr) or decadic logarithm of drug concentration. * or # indicate *p* < 0.05 vs. Ctr or 100 nM GIP, respectively. Numbers in brackets are number of experiments

In contrast to mouse preparations, in human atrium, when GIP was applied alone, GIP concentration- and time-dependently enlarged FOC. This PIE to GIP is visualized in an original recording (Fig. 4A). Like in mouse left atrium, the PIE of GIP in human atrium was attenuated by the GIP-antagonist ProGIP (Fig. 4B). We have collected several experiments in Fig. 4C. We show that GIP lifted FOC when plotted in mN (Fig. 4C).

**Fig. 5:**
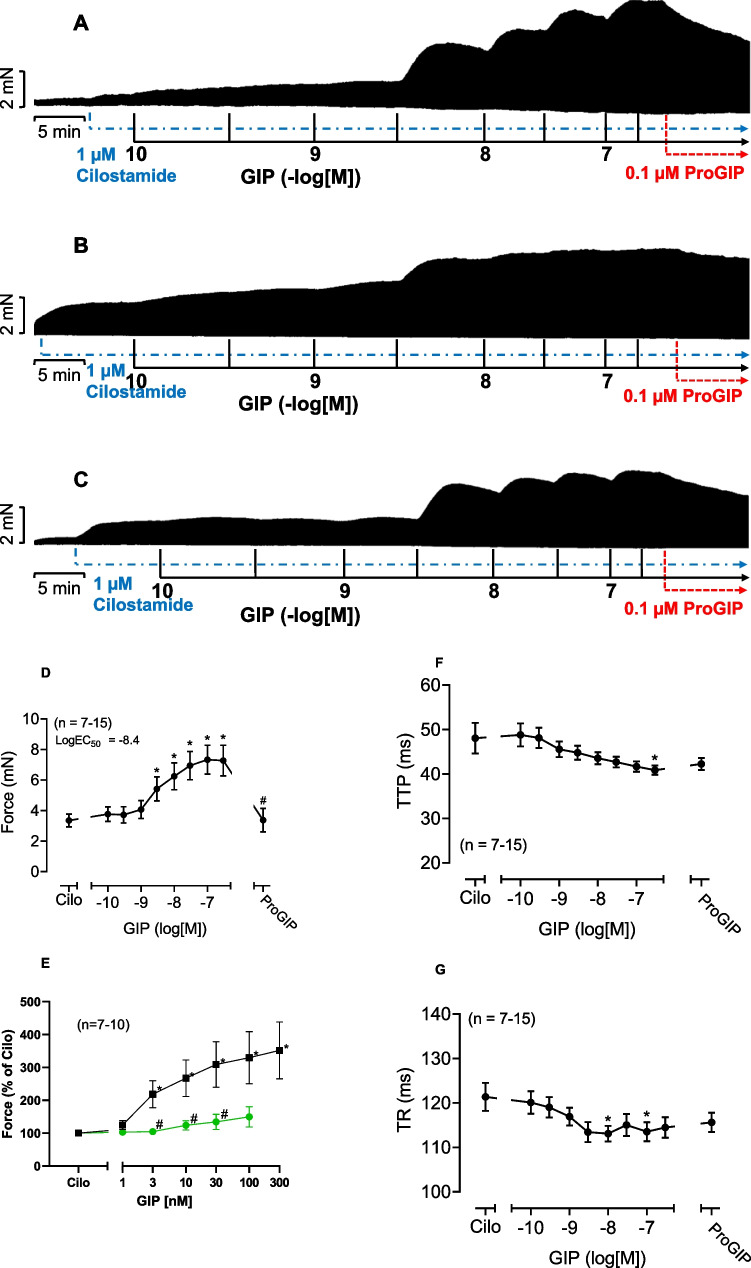
GIP increased force of contraction more potently and effectively in the presence of cilostamide. Original recordings in three different isolated human right atrial preparations. First cilostamide and then GIP were given to increase force of contraction (**A**, **B**, **C**). Cilostamide (1 µM) induced a time-dependent positive inotropic effect (**A–C**). Subsequently, GIP was cumulatively applied and finally 0.1 µM ProGIP was given (**A–C**). Ordinates indicate force in milli Newton (mN). Vertical arrows indicate time of drug addition and horizontal bars indicate time in minutes (min). Summarized effects: force of contraction in mN (**D**), force of contraction in percentage of the effect of cilostamide (Cilo, **G**), time to peak tension (**E**) and time of relaxation (**F**). In **E**, in separate experiments, the effects of cumulatively applied GIP in the presence of only cilostamide (squares) or in the additional presence of 100 nM ProGIP and cilostamide (circles) were plotted. * or # indicate *p* < 0.05 vs. Ctr (after cilostamide). In **G**, # indicates *p* < 0.05 vs. corresponding values without ProGIP. Numbers in brackets indicate the number of experiments

Next, we studied a putative interaction of GIP and cilostamide. Cilostamide per se, a phosphodiesterase III-inhibitor (Fig. 1), raised force of contraction in human atrium. Typical recordings for the effect of GIP are depicted in Fig. 5A. We chose a low concentration of cilostamide that raised nevertheless the FOC (Fig. 5A–C) based on our previous work (e.g. Gergs et al. 2023).

**Fig. 6:**
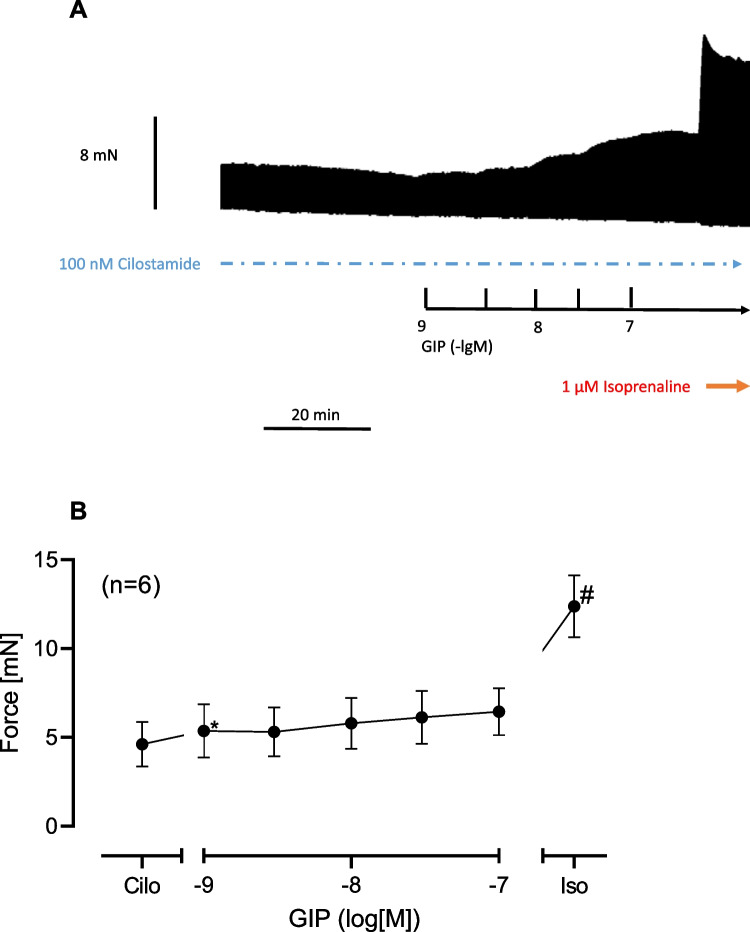
GIP increased force of contraction less effectively than isoprenaline in the presence of cilostamide. Original recording in one isolated human right atrial preparation. First cilostamide and then cumulatively GIP were given to increase force of contraction (**A**). Finally, 1 µM isoprenaline, a β-adrenoceptor agonist, was added to the organ bath (**A**). Long vertical bar indicates force of contraction in mN. Small vertical bars indicate time of GIP addition. Horizontal bar indicates time scale in minutes (min). Horizontal arrow shows where isoprenaline was applied. The negative decadic logarithm of the molar concentration of applied GIP is supplied above the large horizontal arrow. The broken horizontal line indicates the continuous presence of 100 nM cilostamide in the organ bath. Summarized effects: force of contraction in mN (**B**). In **B**, * and # indicate *p* < 0.05 vs. pre-drug GIP value (after addition of cilostamide, Cilo) or 100 nM GIP, respectively. Number in brackets indicates the number of experiments. Ordinate indicates force of contraction in milli Newton (mN)

When we gave first cilostamide to the organ bath to slightly raise force of contraction and then added GIP, GIP enhanced FOC more in the presence of cilostamide than in the absence of cilostamide. This is depicted in a typical original experiment in Fig. 5A. In other words, cilostamide shifted the PIE of GIP to lower concentrations (sinistrally, *p* < 0.05) which can be seen in comparing the EC50 values in Figs. 5D and 4B. Moreover, the effect of GIP in the presence of cilostamide could be reduced by a GIPR antagonist (Fig. 5A–C). We summarized several experiments that confirmed that GIP, in the presence of cilostamide, increased in a concentration- and time-dependent manner FOC in mN (Fig. 5D). In addition, ProGIP (100 nM) subsequently applied reduced FOC as seen in Fig. 5D. GIP abbreviated the time to peak tension (Fig. 5F), and this effect was not antagonized by ProGIP (Fig. 5F). Moreover, GIP also reduced the time of relaxation (Fig. 5G). These effects on time of relaxation were also not antagonized by ProGIP (Fig. 5G). Moreover, as a further control we first gave cilostamide, then added 100 nM ProGIP and then subsequently gave cumulatively GIP. It turned out that ProGIP attenuated the PIE of GIP downwards, suggesting further an antagonism (Fig. 5G). In other experiments, we first applied cilostamide, the added cumulatively GIP and finally applied 1 µM isoprenaline (Fig. 6A). It turned out that GIP was less effective than isoprenaline to raise FOC under these conditions (Fig. 6A). Several experiments were summarized in Fig. 6B that confirmed these findings. Finally, in separate experiments we first gave 100 nM GIP to increase FOC in HAP (by 87%). Then when this effect had reached plateau, we added 1 µM propranolol (a β-adrenoceptor antagonist). This concentration of propranolol slightly reduced the GIP-elevated FOC in HAP, but only 8% of the positive inotropic effect of GIP in HAP (*n* = 3, *p* < 0.05), suggesting a slight contribution of β-adrenoceptor-mediated effects for the PIE of GIP in the human atrium (about 10%).

## Discussion

The main new findings were that GIP augmented FOC in human atrium via GIPR. The fact that we measured a PIE on both mouse left atrium and human atrium concurs with a direct action of GIP in the mammalian heart. When we started this project, it was already known that GIPR are expressed in mouse heart and human heart (vide supra). However, it was unknown whether the GIPR in mouse heart or in human heart were functional, in other words could be stimulated by GIP. This clearly was the case, judged from our present data. Moreover, we could show that these effects are reversible upon washout (data not shown) and are probably GIPR mediated using antagonists at GIPR.

Moreover, we assume that GIP acts, at least in part, via cAMP in human atrium. For one, 10 nM GIP increased intracellular cAMP levels in mouse cardiomyocytes (Hiromura et al. 2016). The effects of GIP on FOC in HAP are amplified by pre-treatment with cilostamide, a PDE 3 inhibitor. These conclusions are further supported by our functional data. Isoprenaline is well known to increase rate of relaxation and accelerate the time of muscle relaxation in the mammalian heart including the human heart by cAMP-dependent phosphorylation of phospholamban (Fig. 1). We noticed the same with GIP: increase in rate of relaxation and shorter time of relaxation. Hence, also in this regard isoprenaline, known to act via cAMP, and GIP, studied here, acted identically suggesting they use similar if not identical signal transduction pathways but other receptors. Of note, ProGIP failed to reversed this shortening (Fig. 5F, G) perhaps due to different kinetics of decrease in cAMP in different compartments of the cell (Fig. 1), but this was not explored further in the present work (Subramaniam et al. 2023).

The fact that we failed to detect a positive chronotropic effect of GIP in mouse right atrium is consistent with the observation by others that in mice with ablation of the GIPR the basal beating rate in these mice was unaltered (Ussher et al. 2018). Hence, there might be cellular heterogeneity of the expression of the GIPR. The GIPR might be expressed in the left atrium of the mouse heart but not in the sinus node of the mouse heart. Based on these observations in mice, it might be fruitful to test whether the GIPR is expressed in the human sinus node. If we assume that mouse and man in this regard behave similarly, then we might hypothesize that the increase in beating rate seen in studies in human patients when GIP was applied is not directly mediated in the human sinus node but via central mechanisms.

Relevant in our context might be that vascular endothelial cells express GIPR. Their stimulation can lead to cAMP generation but also to the release of nitric oxide and endothelin 1, both of which might alter force in surrounding cardiomyocytes (Zhong et al. 2000, Ding et al. 2004). Hence, our data cannot prove that the PIE to GIP in human atrium is caused by GIPR in cardiomyocytes. To resolve this issue, experiments with isolated human cardiomyocytes will be needed in subsequent studies.

Plasma concentrations of GIP in humans before and after typical meals were reported as 5–20 pM and 100–150 pM (Vollmer et al. 2008). In the very same plasma samples, GIP raised GLP-1 and insulin concentrations from 15 to about 40 pM and from 10 to about 100 mU/l, respectively; glucagon concentrations fell from 12 to 6 pM (Vollmer et al. 2008). This means that these GIP concentrations are physiologically active in humans. In some experimental studies much higher concentrations of GIP were used. For instance, in CHO cells stably transfected with DNA for the GIPR, 1 µM GIP was used to increase cAMP levels (even in the additional presence of IBMX) (Kubota et al. 1997). In other experiments with HL-1 (a tumor line) atrial cardiomyocytes from rats, 1 nM GIP failed to increase cAMP levels; instead, 10 nM (the next higher concentration studied) increased cAMP levels (Ussher et al. 2018). Others, like we used also nanomolar and not picomolar concentrations of GIP. For example, phosphorylation of CREB, a substrate of PKA, or production of arachidonic acid via phospholipase A2 or activation of ERK or inhibition of potassium currents or phosphorylation of PKB or phosphorylation of Foxo1 or increase in free cytosolic Ca^2+^ gained significance in cell culture studies (using e.g. INS-1 β cells) at 0.1–1 nM GIP and higher concentrations of GIP (Bollag et al. 2000; Ehses et al. 2001; 2002, Kim et al. 2005a; 2005b; Kim et al. 2008; Trümper et al. 2001). In experiments human endothelial cells GIP concentrations of 100 nM or more started to induce significant changes in measured contractile parameters (Mori et al. 2018).

Under certain conditions quite high concentrations of GIP may be reached in humans, for instance if metabolism of GIP is impaired. For instance, dipeptidyl peptidase-4 (DDP-4) degrades and inactivates GIP. Therefore, inhibitors of DDP-4 like sitagliptin increase plasma levels of GIP in patients, and thus higher plasma levels of GIP occur in some patients more similar to the concentrations we used here (Idorn et al. 2014). Hence, our concentrations used are higher than those measured in vivo in plasma but are in line with the concentrations used by others in the organ bath.

### Clinical relevance

GIP (42 amino acids) is formed by endogenous proteases from its precursor pre-pro-GIP (153 amino acids, Lindquist et al. 2022). The specific mRNA for pre-pro-GIP is mainly found in the gut (Lindquist et al. 2022). Hence, as we noted PIE of GIP started with 1 nM (with cilostamide present), we argue the physiological concentrations of GIP can already stimulate the human heart. There are studies with proteolysis-resistant mutants of GIP that may be used in the future as drugs (Heimbürger et al. 2020). From our data we predict that these stable derivatives of GIP will also activate GIPR in the human heart. There exists a statistical positive correlation between cardiovascular disease and plasma levels of GIP (Kahles et al. 2018; Hiromura et al. 2021). This does not prove causality but suggests also the GIP might be beneficial or detrimental in cardiovascular diseases, but this warrants further work because even in animal studies controversy on the role of GIP in this regard exists.

Tumors can produce GIP and increase GIP concentrations in the plasma of patients (Faria et al. 2022; Regazzo et al. 2022; Velikyan et al. 2022; Refardt et al. 2022; Gourni et al. 2014; Lacroix 2023). Moreover, one would predict that tumors that produce GIP can be companied by cardiovascular effects like atrial fibrillations, because GIP can increase cAMP in cardiomyocytes and all cAMP-increasing agents increase the incidence of atrial fibrillation and other arrhythmias in patients (e.g. Beneke and Molina 2021). These side effects might be mediated by direct cardiac effects of GIP from tumors on GIPR. Therefore, these tumors might be treated by radioactive GIPR antagonists. We speculate that this treatment of the GIP producing tumors should end atrial fibrillations.

In summary, we present evidence for a positive inotropic effect of GIP via GIPR in human atrium.

## Data Availability

All source data for this work (or generated in this study) are available upon reasonable request.
